# Artificial intelligence detection of heart failure on coronary computed tomography angiography: external validation in patients with non-ST-segment elevation acute coronary syndrome

**DOI:** 10.1093/ehjdh/ztag133

**Published:** 2026-09-01

**Authors:** Anne Sophie Overgaard Olesen, Kristina Cecilia Miger, Silas Nyboe Ørting, Jens Petersen, Marleen de Bruijne, Mikael Ploug Boesen, Klaus Fuglsang Kofoed, Johannes Grand, Jens Jakob Thune, Alasdair D Henderson, Pardeep S Jhund, Lars Køber, Olav Wendelboe Nielsen

**Affiliations:** Department of Cardiology, Copenhagen University Hospital—Bispebjerg and Frederiksberg, Bispebjerg Bakke 23, 2400 Copenhagen, Denmark; Department of Clinical Medicine, University of Copenhagen, Blegdamsvej 3B, 2200 Copenhagen, Denmark; British Heart Foundation Cardiovascular Research Centre, School of Cardiovascular and Metabolic Health, University of Glasgow, 126 University Place, G12 8TA Glasgow, UK; Department of Cardiology, Copenhagen University Hospital—Bispebjerg and Frederiksberg, Bispebjerg Bakke 23, 2400 Copenhagen, Denmark; Department of Computer Science, University of Copenhagen, Copenhagen, Denmark; Department of Computer Science, University of Copenhagen, Copenhagen, Denmark; Department of Computer Science, University of Copenhagen, Copenhagen, Denmark; Department of Radiology and Nuclear Medicine, Erasmus University Medical Center, Rotterdam, Netherlands; Department of Clinical Medicine, University of Copenhagen, Blegdamsvej 3B, 2200 Copenhagen, Denmark; Department of Radiology, Copenhagen University Hospital—Bispebjerg and Frederiksberg, Copenhagen, Denmark; Department of Clinical Medicine, University of Copenhagen, Blegdamsvej 3B, 2200 Copenhagen, Denmark; Department of Cardiology, Copenhagen University Hospital—Rigshospitalet, Copenhagen, Denmark; Department of Radiology, Copenhagen University Hospital—Rigshospitalet, Copenhagen, Denmark; Department of Cardiology, Copenhagen University Hospital—Amager and Hvidovre, Hvidovre, Denmark; Department of Cardiology, Copenhagen University Hospital—Bispebjerg and Frederiksberg, Bispebjerg Bakke 23, 2400 Copenhagen, Denmark; Department of Clinical Medicine, University of Copenhagen, Blegdamsvej 3B, 2200 Copenhagen, Denmark; British Heart Foundation Cardiovascular Research Centre, School of Cardiovascular and Metabolic Health, University of Glasgow, 126 University Place, G12 8TA Glasgow, UK; British Heart Foundation Cardiovascular Research Centre, School of Cardiovascular and Metabolic Health, University of Glasgow, 126 University Place, G12 8TA Glasgow, UK; Department of Clinical Medicine, University of Copenhagen, Blegdamsvej 3B, 2200 Copenhagen, Denmark; Department of Cardiology, Copenhagen University Hospital—Amager and Hvidovre, Hvidovre, Denmark; Department of Cardiology, Copenhagen University Hospital—Bispebjerg and Frederiksberg, Bispebjerg Bakke 23, 2400 Copenhagen, Denmark

**Keywords:** Artificial intelligence, Coronary CT angiography, Heart failure, NSTE-ACS, Congestion, Acute coronary syndrome

## Abstract

**Aims:**

Heart failure (HF) in non-ST-segment elevation acute coronary syndrome (NSTE-ACS) is associated with poor prognosis but often under-recognized. Coronary computed tomography angiography (CCTA), increasingly used in NSTE-ACS, contains cardiopulmonary features not routinely assessed for HF. We evaluated whether an artificial intelligence (AI) algorithm applied to CCTA could identify HF likelihood in NSTE-ACS.

**Methods and results:**

In this retrospective external validation study, the AI algorithm was applied without retraining or recalibration to CCTA scans from 1009 patients with NSTE-ACS in the VERDICT trial. Using a pre-specified threshold, patients were classified as low or high AI likelihood of HF. The primary outcome was HF during index hospitalization. The secondary outcome was post-discharge HF hospitalization among patients discharged alive without HF, with analyses adjusted for global registry of acute coronary events score >140 and severe coronary artery disease. Death was treated as a competing risk. Overall, 838 patients (83%) were classified as low AI likelihood and 171 (17%) as high. During index hospitalization, HF was diagnosed in 10 patients (1%) with low AI likelihood and 12 (7%) with high. Sensitivity was 55%, specificity 84%, positive predictive value 7%, and negative predictive value 99%. High AI likelihood was associated with increased risk of index HF (subdistribution hazard ratio, 5.39, 95% confidence interval (CI) 2.32–12.50). After discharge, HF hospitalization occurred in 25 patients (3%) with low AI likelihood and 14 (8%) with high. High AI likelihood remained associated with HF hospitalization (subdistribution hazard ratio 2.56, 95% CI 1.34–4.90).

**Conclusion:**

AI-based CCTA analysis identified a large low-risk subgroup and a smaller subgroup at increased HF risk, supporting further evaluation of opportunistic HF assessment from CCTA.

## Graphical Abstract

**Graphical Abstract:**
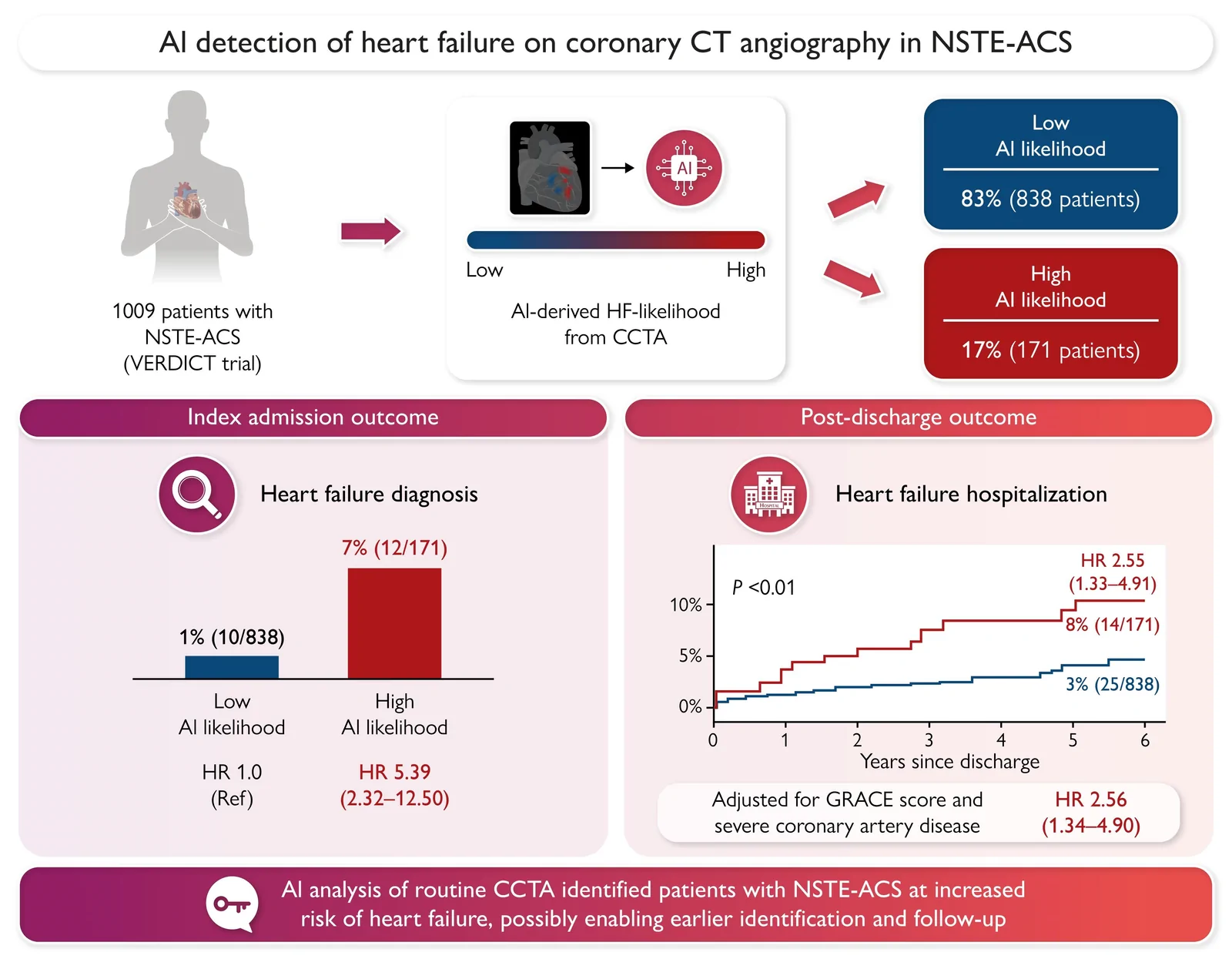

## Introduction

Heart failure (HF) complicating non-ST-segment elevation acute coronary syndrome (NSTE-ACS)^1^ is associated with poor prognosis and warrants early recognition to enable timely therapy and closer clinical surveillance.^2^ However, HF may be under-recognized in NSTE-ACS, as clinical signs can be subtle, transient, or masked by concomitant ischaemia.^3,4^

Coronary computed tomography angiography (CCTA) is increasingly used in the diagnostic workup of suspected or confirmed NSTE-ACS. Beyond coronary anatomy, CCTA contains extracardiac and cardiac features associated with HF, including pulmonary congestion, pleural effusions, cardiac chamber enlargement, and venous congestion.^5^ These features are not routinely incorporated into clinical decision-making, partly because they lack standardized definitions and systematic assessment.^2^ Observational studies have shown that pulmonary congestion and quantitative lung attenuation measures on CCTA correlate with cardiac dysfunction and predict subsequent HF hospitalization and mortality.^6,7^

Artificial intelligence (AI) may enable standardized extraction of HF-related imaging features from computed tomography (CT). We previously developed an interpretable AI algorithm for CT-based detection of HF^8^ and subsequently validated it in patients undergoing chest CT for acute dyspnoea.^9^ Whether this AI-based assessment can provide diagnostic and prognostic information when applied to routinely acquired CCTA scans in patients with NSTE-ACS, and how such information relates to existing clinical risk assessment, such as global registry of acute coronary events (GRACE) score and coronary anatomy,^10^ remains unknown. We therefore aimed to externally validate the algorithm for identifying HF during index hospitalization and to assess its association with subsequent HF hospitalization after discharge, with death treated as a competing event.

## Methods

### Study design

This retrospective external validation study evaluated a previously developed AI algorithm for CT-based likelihood of HF^8^ when applied to CCTA in patients with NSTE-ACS, in accordance with a pre-specified development and validation framework.^11^

### Validation cohort: the VERDICT trial

The validation cohort was derived from the VERDICT trial (very early vs. deferred invasive evaluation using computerized tomography; ClinicalTrials.gov NCT02061891), a prospective, multicentre, randomized trial of 2147 patients with confirmed NSTE-ACS and at least one high-risk criterion, randomized to invasive evaluation within 12 h or within 48–72 h.^12^ In a pre-specified observational substudy, clinically blinded CCTA was performed during index hospitalization, after randomization and before invasive coronary angiography, in 1023 patients.^13^ Coronary computed tomography angiography was acquired using prospective electrocardiographic (ECG)-triggering and intravenous contrast on 64- or 320-detector scanners according to local protocols.

### Development of the artificial intelligence algorithm

The AI algorithm has been described previously.^8^ In brief, the algorithm was developed in a single-centre, retrospective cohort of 4672 patients who underwent acute chest CT for any clinical indication from undifferentiated medical departments at Copenhagen University Hospital—Bispebjerg and Frederiksberg, Denmark, between 2016 and 2021. The diagnostic standard for model development consisted of radiological evidence of acute HF and pulmonary congestion, derived through structured text-mining of radiology reports. Automated anatomical segmentation was performed using TotalSegmentator,^14^ and prediction was implemented using a tree-based gradient boosting model (XGBoost).^15^ During model development, the cohort was randomly split on subject level, with two-thirds used for training and one-third held out as an independent test set for internal validation.

The AI model outputs a continuous score ranging from 0 to 1, with higher scores reflecting higher likelihood of HF. The model integrates 12 features derived from automated cardiopulmonary segmentation: mean lung boundary density, median lung density, pleural effusion volume, pleural ratio (pleural effusion volume relative to total lung volume), left and right atrial volumes, right ventricular ratio (right ventricular volume relative to total heart volume), absolute heart Z-score (standardized total heart volume), inferior vena cava diameter, mean density of the inferior vena cava, right atrial density, and patient age. Three thresholds were pre-specified during internal validation: an optimal Youden threshold, a rule-out threshold (sensitivity ≥90%), and a rule-in threshold (specificity ≥90%).

### Artificial intelligence-based detection of HF in VERDICT scans and definition of likelihood groups

The AI algorithm was applied to VERDICT CCTA scans without retraining, recalibration, or modification. Because CCTA provides more limited lung coverage than full chest CT, a preliminary analysis was performed in the original development dataset before access to VERDICT scans. Development chest CT scans were truncated to the cardiac extent, and model discrimination was compared using full-coverage, reduced-coverage, and regression-corrected reduced-coverage features. Discrimination was similar across approaches, and VERDICT CCTA scans were therefore analysed without feature correction to preserve the external validation design. For the main analyses, patients were categorized using the pre-specified rule-out threshold into low AI-derived likelihood or high AI-derived likelihood of HF. A three-group stratification using the pre-specified rule-out and Youden threshold is presented in the Supplementary material.

### Study outcomes and event definitions

The primary outcome was HF diagnosed during the index hospitalization, adjudicated by study investigators based on medical record review blinded to CCTA findings. Heart failure was defined as a clinically documented diagnosis during admission based on available information in the medical record, including physician documentation, clinical course, treatment during admission, and available cardiac investigations. The AI-derived likelihood score was generated retrospectively and was not available during clinical care or outcome adjudication.

Time 0 was defined as the date of CCTA, and patients were followed until hospital discharge. Because some patients were transferred from referring hospitals before CCTA, HF could occasionally be diagnosed before imaging. Exact event times were unavailable, and timing was therefore based on calendar dates. The CCTA date was defined as Day 0, and diagnoses recorded on either of the two preceding calendar dates were accepted as events at Time 0. This window was chosen to allow for short clinical and administrative delays while maintaining temporal proximity to imaging. In addition, imaging features of HF were considered likely to remain detectable for a short period after diagnosis, including after initiation of treatment. Earlier diagnoses were not counted as index-HF events, although these patients remained in the cohort and contributed follow-up time for mortality.

The secondary outcome was HF hospitalization after discharge, identified using discharge diagnosis codes. Follow-up began at hospital discharge and continued until the end of follow-up. Heart failure was analysed as a first-event outcome. Accordingly, patients with diagnosed HF during the index hospitalization had already experienced a HF event and were therefore not considered at risk for a first HF hospitalization after discharge. These patients remained in the cohort and continued to contribute follow-up for death, which was treated as a competing event. Median follow-up from hospital discharge was 4.8 years [inter-quartile range (IQR) 3.7–5.8 years].

### Statistical analyses

Baseline characteristics were summarized using mean (standard deviation), median (IQR), or counts (percentages), as appropriate. In two supplementary analyses, baseline characteristics were stratified according to HF occurrence during the study course and according to AI-derived HF likelihood. Between-group comparisons were performed using Student’s *t*-test or Wilcoxon rank-sum test for continuous variables and χ^2^ or Fisher’s exact test for categorical variables, as appropriate.

For the primary diagnostic analyses during the index hospitalization, HF diagnosed from the time of CCTA until hospital discharge was analysed with death treated as a competing event. Associations between AI-likelihood groups and outcomes were estimated using Fine–Gray regression and reported as subdistribution hazard ratios (sHRs). Diagnostic performance was assessed using sensitivity, specificity, predictive values, likelihood ratios, and receiver operating characteristic (ROC) analyses with area under the curve (AUC).

Post-discharge analyses used hospital discharge as a landmark time point. Heart failure hospitalization after discharge was analysed using Fine–Gray regression with death treated as a competing event. Multivariable models examined the association between AI-derived likelihood and post-discharge HF hospitalization after adjustment for GRACE score >140 and severe coronary artery disease, defined as three-vessel or left main disease on invasive coronary angiography.^12^ A second model additionally included sex, hypertension, and diabetes. A third exploratory model further included left ventricular ejection fraction (LVEF) and a history of HF. Multivariable analyses were performed using complete-case data without imputation. Exploratory ROC analyses assessed discrimination for post-discharge HF hospitalization at 1 and 5 years using all combinations of AI-derived likelihood, GRACE score >140, and severe coronary artery disease. Confidence intervals (CIs) and *P-*values for incremental AUC changes after adding AI-derived likelihood were estimated using non-parametric bootstrap with 1000 resamples.

All analyses were performed using R (version 4.4.2).

### Ethical considerations and consent

The VERDICT trial was approved by the Danish National Committee on Health Research Ethics and the Danish Data Protection Agency (ID: H-4-2010-039), and all participants provided written informed consent. Development and validation of the AI model were approved by the Danish Research Ethics Committee (ID: 1575037). Data were pseudonymized before researcher access and analysed in accordance with Danish data protection regulations.

## Results

### Baseline characteristics

Of the 1023 patients who underwent CCTA in the VERDICT trial, 14 were excluded from the present analysis: 12 patients for whom CCTA datasets could not be retrieved, and in 2 patients, hospital admission or discharge dates were unavailable, precluding assessment of index-hospitalization length. The final study population therefore comprised 1009 patients.

The mean age of the study population was 61.8 years, and 67% were men (*Table 1*). Hypertension was present in 48% of patients, diabetes in 13%, and a history of prior myocardial infarction in 14%. Previously diagnosed HF was documented in 9% of patients. During the index hospitalization, the median LVEF was 55%, and the invasive coronary angiography demonstrated three-vessel or left main coronary artery disease in 15% of patients. Elevated troponin concentrations at admission, defined as values above the assay-specific upper reference limit used at each participating centre, were observed in 77%, and 43% had a GRACE score >140.

**Table 1:** Baseline characteristics

	Overall (*N* = 1009)	Missing, *n* (%)
Patient characteristics		
Sex male, *N* (%)	677 (67)	0 (0)
Age (years), mean (SD)	61.8 (11.9)	0 (0)
BMI (kg/m^2^), median [IQR]	26.3 [24.0, 29.3]	6 (1)
History of heart failure, *N* (%)	95 (9)	0 (0)
History of myocardial infarction, *N* (%)	145 (14)	0 (0)
History of diabetes, *N* (%)	130 (13)	0 (0)
History of hypertension, *N* (%)	487 (48)	0 (0)
History of chronic obstructive pulmonary disease, *N* (%)	130 (13)	0 (0)
History of renal disease, *N* (%)	60 (6)	0 (0)
Current smoker, *N* (%)	339 (34)	0 (0)
Admission data		
Systolic blood pressure (mmHg), mean (SD)	141 (22)	8 (1)
Heart rate, median [IQR]	72 [62–84]	12 (1)
Atrial fibrillation at admission, *N* (%)	33 (3)	0 (0)
Increased troponins, *N* (%)^a^	781 (77)	10 (1)
Creatinine (μmol/L), median [IQR]	76 [66, 88]	13 (1)
LVEF at admission (%), median [IQR]	55 [50, 60]	141 (14)
Revascularization during admission, *N* (%)	621 (62)	0 (0)
Three-vessel or left main coronary artery disease, *N* (%)^b^	154 (15)	0 (0)
GRACE score >140, *N* (%)	431 (43)	17 (2)
Length of index stay, median [IQR]^c^	2 [1–4]	0 (0)
Treatment at discharge of admission		
Aspirin, *N* (%)	866 (86)	4 (0)
P2Y12 receptor inhibitor, *N* (%)	708 (70)	6 (1)
Oral anticoagulant, *N* (%)	38 (4)	5 (1)
Statin, *N* (%)	864 (86)	15 (2)
Beta-blocker, *N* (%)	714 (71)	14 (1)
ACEi and/or ARB, *N* (%)	358 (36)	18 (2)
Loop diuretics, *N* (%)	88 (9)	15 (2)
MRA, *N* (%)	32 (3)	16 (2)

^a^Elevated troponin was defined as a value above the assay-specific upper reference limit, consistent with the VERDICT trial.

^b^Three-vessel or left main coronary artery disease was assessed by invasive coronary angiography performed during the index hospitalization.

^c^Total days from admission at invasive centre or primary hospital to the final hospital discharge.

### Outcomes

Median time from CCTA to hospital discharge was 1 day, and 88% of patients were discharged within 48 h. Because CCTA was not always performed on the admission day, 3 patients had HF diagnosed outside the pre-specified time window, at 4, 5, and 7 days before CCTA, and were therefore not counted as index-HF events.

During the index hospitalization, HF was diagnosed in 22 patients (2%) within the pre-specified time window anchored at CCTA, and 1 patient (0.1%) died before discharge without a preceding HF diagnosis. From discharge to the end of follow-up, 39 patients (4%) experienced a first HF hospitalization, and 105 patients (10%) died (*Table 2*; Supplementary material online, *Figures S1 and S2*). Among patients with post-discharge HF hospitalization, the median time from hospital discharge to first HF hospitalization was 1.4 years (IQR 0.3–3.2 years).

**Table 2:** Clinical outcomes following coronary computed tomography angiography at pre-specified time intervals

	Index hospitalization	Post-discharge		
*N* = 1009	Time 0-discharge, *n* (%)	Discharge-Day 30, *n* (%)	Discharge-1 year, *n* (%)	Discharge-end of follow-up, *n* (%)
Heart failure diagnosis or hospitalization	22 (2)	5 (0.5)	15 (2)	39 (4)
All-cause mortality	1 (0.1)	1 (0.1)	20 (2)	105 (10)

Time 0 was defined as the date of coronary computed tomography angiography. Post-discharge outcomes were counted from hospital discharge. Heart failure was counted as a first event only, so patients with heart failure during index hospitalization were not counted again for post-discharge heart failure. Median follow-up from discharge was 4.8 years (IQR 3.7–5.8).

Baseline characteristics according to HF occurrence during the study course are presented in Supplementary material online, *Table S2*. Patients who experienced HF during the index hospitalization or after discharge were older and more frequently had a history of HF, myocardial infarction, and renal disease. Furthermore, they exhibited lower LVEF, higher heart rates and creatinine levels at admission, and more frequent use of loop diuretics, angiotensin-converting enzyme inhibitors (ACEi) and/or angiotensin II receptor blockers (ARBs), and mineralcorticoid receptor antagoinists (MRAs) at discharge.

### Comparison of artificial intelligence likelihood groups with heart failure diagnosed during index hospitalization

Overall, 838 patients (83%) were classified as having low AI-derived likelihood of HF, and 171 (17%) as having high AI-derived likelihood using the pre-specified rule-out threshold (*Table 3*). Patients with high AI likelihood were older, had lower LVEF, and more frequently had a GRACE score >140 and were treated with loop diuretics at discharge compared with those with low likelihood (see Supplementary material online, *Table S3*).

**Table 3:** Association between artificial intelligence-derived likelihood and heart failure diagnosis and hospitalizations

Heart failure diagnosis during index hospitalization				
AI HF likelihood	*n*	HF diagnosis, *n* (%)	All-cause mortality (before HF diagnosis), *n* (%)	sHR (95% CI)
**Low likelihood**	838	10 (1)	1 (0.1)	Ref
**High likelihood**	171	12 (7)	0	5.39 (2.32–12.50)

Heart failure hospitalization after discharge				
AI HF likelihood	*N*	HF hospitalization, *n* (%)	All-cause mortality (after discharge, before HF hospitalization), *n* (%)	sHR (95% CI)
**Low likelihood**	838	25 (3)	61 (7)	Ref
**High likelihood**	171	14 (8)	26 (15)	2.55 (1.33–4.91)

For the post-discharge analysis, hospital discharge was used as the landmark time point. Patients diagnosed with heart failure during the index hospitalization were not counted as being at risk for a subsequent post-discharge heart failure hospitalization, but they remained under follow-up for death.

From Time 0 until hospital discharge, HF was diagnosed in 10 (1% of 838) patients with low AI likelihood and in 12 (7% of 171) patients with high AI likelihood. One death occurred in the low-likelihood group, and none in the high-likelihood group. Using high AI likelihood as a positive test result for HF during index hospitalization, sensitivity was 55% (95% CI 32–76), specificity 84% (95% CI 81–86), positive predictive value 7% (95% CI 4–12), and negative predictive value 99% (95% CI 98–99). The positive and negative likelihood ratios were 3.39 (95% CI 2.25–5.09) and 0.54 (95% CI 0.34–0.86), respectively, and the AUC was 0.69 (95% CI 0.59–0.80).

High AI likelihood was associated with a HF diagnosis during the index hospitalization compared with low likelihood (sHR 5.39, 95% CI 2.32–12.50; *P* < 0.001) (*Table 3*). In supplementary analyses using the three pre-specified AI-likelihood groups (low, intermediate, and high), a graded increase in risk of HF during the index hospitalization was observed (see Supplementary material online, *Table S4*).

### Post-discharge heart failure hospitalizations across artificial intelligence likelihood groups

**Figure 1:**
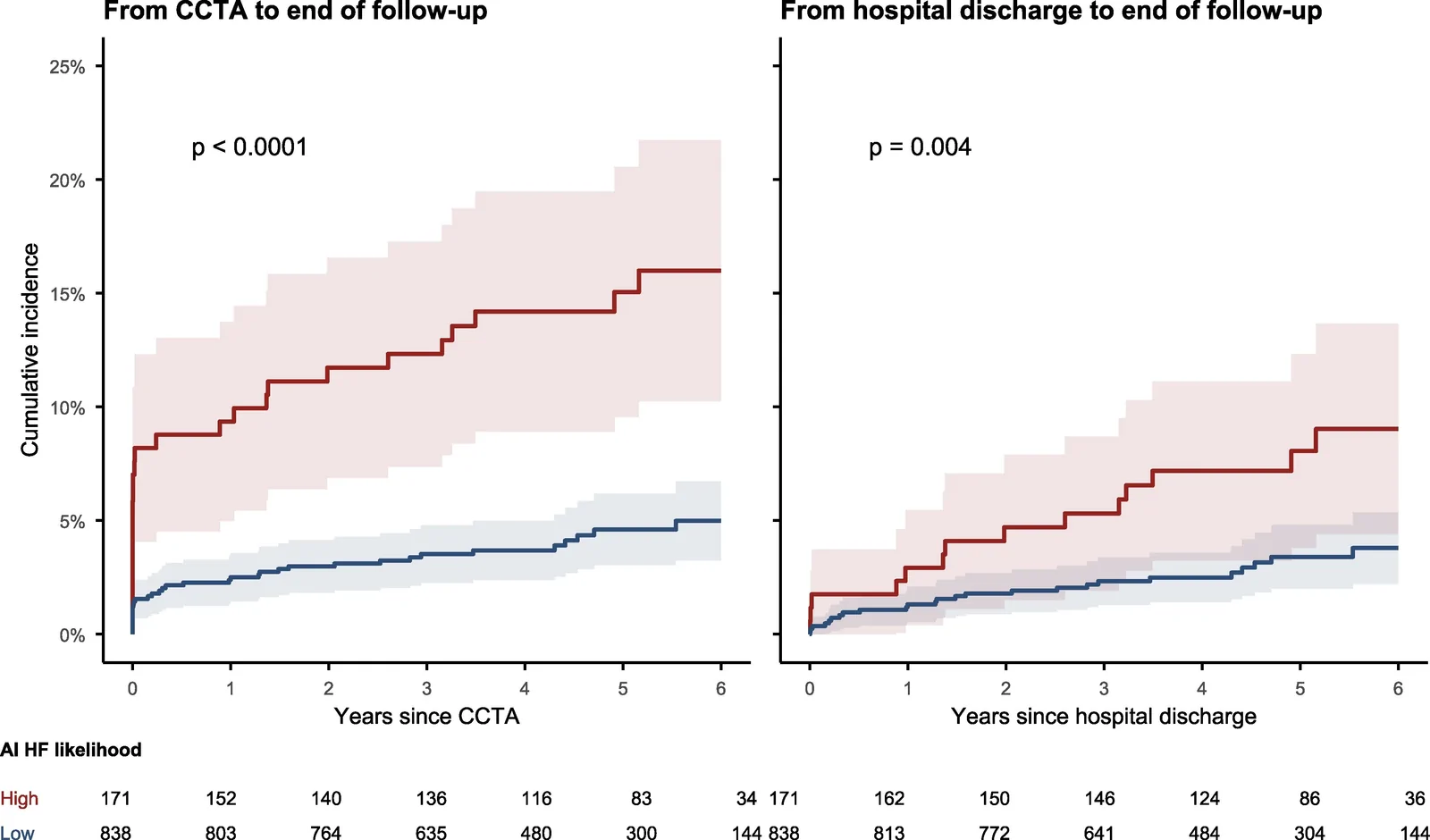
Cumulative incidence of heart failure hospitalization according to AI-derived likelihood. Cumulative incidence functions for heart failure diagnosis or hospitalization are shown from coronary computed tomography angiography to end of follow-up and from hospital discharge to end of follow-up, stratified by low and high artificial intelligence-defined heart failure likelihood. Death was treated as a competing event. Shaded areas indicate 95% confidence intervals. Numbers below the plots indicate patients at risk.

In the discharge landmark analysis, high AI-derived likelihood of HF was associated with a higher incidence of post-discharge HF hospitalization than low likelihood (sHR 2.55, 95% CI 1.33–4.91) (*Table 3*). Cumulative incidence curves in the post-discharge landmark analysis also showed separation between the two groups (*Figure 1*). In a sensitivity analysis from Time 0, cumulative incidence curves likewise showed separation throughout the entire follow-up period (*Figure 1*). Supplementary analyses using the three AI-likelihood groups showed similar overall patterns (see Supplementary material online, *Table S4* and *Figure S3*).

### Adjusted association between artificial intelligence-derived likelihood groups and post-discharge heart failure hospitalization

After adjustment for GRACE score >140 and three-vessel or left main coronary artery disease, high AI-derived likelihood of HF remained associated with post-discharge HF hospitalization (sHR 2.56, 95% CI 1.34–4.90) (*Table 4*). Similar estimates were observed after additional adjustment for sex, hypertension, and diabetes (sHR 2.52, 95% CI 1.32–4.80) (*Table 4*). In a third exploratory model additionally including LVEF and a history of HF (*n* analysed = 853), each 10 percentage-point decrease in LVEF remained associated with post-discharge HF hospitalization (sHR 1.39, 95% CI 1.01–1.91), as did three-vessel or left main coronary artery disease (sHR 2.37, 95% CI 1.01–5.53). The remaining estimates, including those for high AI-derived likelihood (sHR 1.86, 95% CI 0.84–4.10), GRACE score >140 (sHR 1.99, 95% CI 0.91–4.39), and a history of HF (sHR 1.88, 95% CI 0.78–4.56), were attenuated and were not statistically significant.

**Table 4:** Multivariable adjusted association between artificial intelligence-derived likelihood and post-discharge heart failure hospitalization

Variables included in model 1 (*n* analysed = 992, heart failure events = 37, deaths = 80)	sHR	95% CI	*P*-value
**High AI HF likelihood**	2.56	1.34–4.90	0.004
**High GRACE score (>140)**	3.36	1.57–7.19	0.002
**Three-vessel disease or left main coronary artery disease**	2.39	1.20–4.76	0.013

Variables included in model 2 (*n* analysed = 992, heart failure events = 37, deaths = 80)	sHR	95% CI	*P*-value
**High AI HF likelihood**	2.52	1.32–4.80	0.005
**High GRACE score (>140)**	3.32	1.57–7.01	0.002
**Three-vessel disease or left main coronary artery disease**	2.36	1.18–4.73	0.015
**Male sex**	0.84	0.42–1.67	0.620
**Diabetes**	1.55	0.70–3.45	0.280
**Hypertension**	1.47	0.74–2.92	0.270

Multivariable Fine–Gray regression models for post-discharge heart failure hospitalization, with death treated as a competing event. Follow-up began at hospital discharge. Missing data were handled using complete-case analysis for each model.

**Figure 2:**
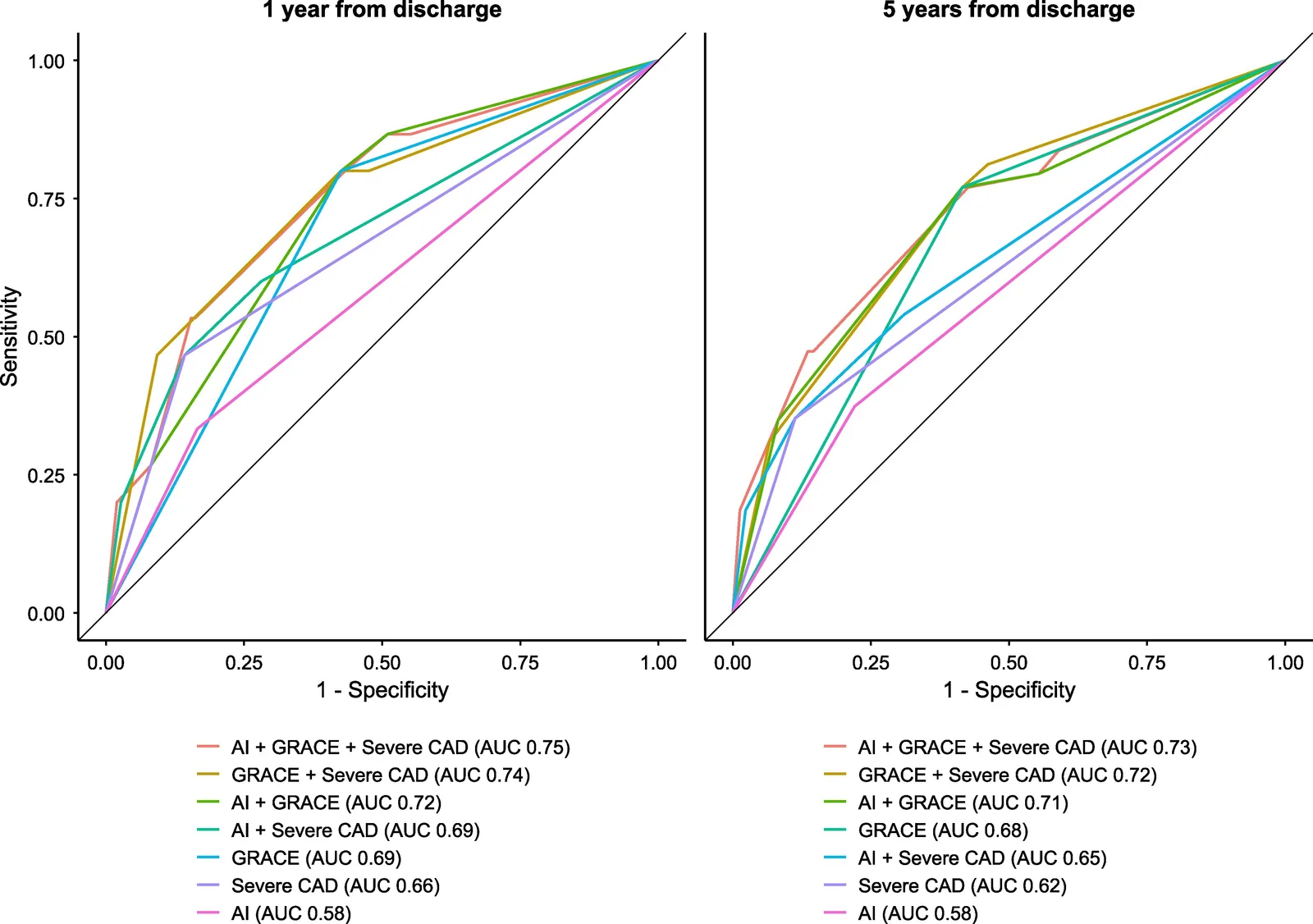
Receiver operating characteristic curves for prediction of incident heart failure at 1 and 5 years. Time-dependent receiver operating characteristic curves for incident heart failure after discharge using artificial intelligence likelihood groups, global registry of acute coronary events score, and coronary artery disease severity, defined as three-vessel disease or left main coronary artery disease, accounting for competing risk of death. Adding artificial intelligence-derived likelihood to global registry of acute coronary events score >140 and severe coronary artery disease did not significantly improve area under the curve at 1 year (ΔAUC 0.01, 95% CI −0.04 to 0.11; *P* = 0.740) or 5 years (ΔAUC 0.01, 95% CI −0.04 to 0.04; *P* = 0.942).

In ROC analyses, the numerically highest AUC was observed for the model including high AI-derived likelihood, GRACE score >140, and severe coronary artery disease (AUC 0.75 at 1 year and 0.73 at 5 years; *Figure 2*). In exploratory incremental discrimination analyses, adding AI-derived likelihood to GRACE score >140 and severe coronary artery disease did not significantly improve AUC at 1 year (ΔAUC 0.01, 95% CI −0.04 to 0.11; *P* = 0.740) or 5 years (ΔAUC 0.01, 95% CI −0.04 to 0.04; *P* = 0.942).

## Discussion

This study provides the first external validation of an AI algorithm for estimating HF likelihood from CCTA in patients with NSTE-ACS. Patients classified as low AI-derived likelihood had a low incidence of HF during the index hospitalization and follow-up. In contrast, patients with high AI-derived likelihood had higher observed risk during hospitalization and after discharge, supporting the proof of concept that AI-derived cardiopulmonary features from routine CCTA are associated with clinically recognized HF outcomes in this population.

AI applications in CCTA have mainly focused on coronary atherosclerosis, including plaque burden, stenosis severity, and functional surrogates, such as CT-derived fractional flow reserve.^16–20^ In contrast, the present study assessed whether AI could extract extracoronary and cardiac features related to HF. Previous observational studies have shown that pulmonary congestion and quantitative lung attenuation measures on CCTA correlate with cardiac dysfunction and predict adverse outcomes.^6,7^ Our findings extend these observations by showing that an externally developed AI algorithm can identify clinically relevant HF patterns from CCTA without retraining or recalibration.

By translating cardiac and extracardiac features into an imaging-based estimate of HF likelihood, AI-assisted CCTA analysis may provide a complementary, structured way to explore HF-related information already present on CCTA. In the present NSTE-ACS cohort, low AI-derived likelihood was associated with a low observed incidence of HF during the index admission, whereas high AI-derived likelihood identified patients at increased risk of post-discharge HF hospitalization, even after adjustment for GRACE score and severe coronary artery disease. This association was attenuated after additional adjustment for LVEF, suggesting that the AI-derived score may partly capture structural or functional cardiac abnormalities related to ventricular dysfunction. In exploratory incremental discrimination analyses, the addition of AI-derived likelihood to GRACE score and severe coronary artery disease resulted in small, non-significant changes in AUC. Future studies should externally validate and refine CCTA-based AI assessment in larger cohorts with more HF events and evaluate its incremental value in combination with natriuretic peptides, echocardiography, and established clinical risk scores.

If validated and refined in CCTA-specific cohorts, integration into the CCTA workflow as an automated background process could support early recognition of cardiopulmonary features relevant to possible HF and inform consideration of natriuretic peptides, echocardiography, monitoring, and discharge planning. The use of SHapley Additive exPlanations (SHAP)-based feature attribution may further support clinical interpretability by providing transparent information on individual risk scores. Future studies should determine whether AI-derived likelihood estimates are sufficiently robust and actionable to influence clinical decision-making, resource utilization, and patient outcomes, and whether performance can be improved through recalibration to CCTA-specific data and integration with established predictors, such as GRACE score and coronary artery disease severity.

The AI algorithm was originally developed on full chest CT scans to detect imaging features of haemodynamic congestion and reflects a continuum of cardiopulmonary abnormalities, including lung attenuation, pleural effusions, cardiac chamber enlargement, and venous congestion.^8^ Applying the algorithm to CCTA introduced technical considerations related to limited lung coverage and contrast enhancement. Coronary computed tomography angiography may truncate pulmonary features, such as peripheral interstitial changes or small pleural effusions, whereas cardiac chamber and central venous features are likely less affected. However, a preliminary analysis in the development dataset showed that reduced scan coverage had little effect on discrimination and that regression-based feature correction did not improve performance. Because the development dataset included both contrast-enhanced and non-contrast scans, attenuation-based features may also differ when applied to consistently contrast-enhanced CCTA. In the present study, the algorithm and pre-specified thresholds were applied without retraining or recalibration to preserve a strict external validation design. The lower sensitivity observed for HF during the index hospitalization indicates that the pre-specified rule-out threshold derived from full chest CT does not directly translate as a clinical rule-out threshold in CCTA. In addition, some HF events included in the index-hospitalization outcome were diagnosed one or two calendar days before CCTA, during which intervening treatment may have reduced imaging signs of congestion at the time of scanning. Future studies should therefore evaluate CCTA-specific recalibration or adaptation of the algorithm and define operating points according to the intended clinical use.

This study has several strengths. It represents a strict external validation of an interpretable AI algorithm applied to a new clinical context, without retraining or recalibration. The algorithm was developed on heterogeneous chest CT data and validated in a separate CCTA cohort, allowing assessment across different scanner and acquisition settings. The analysis was conducted in a well-characterized, multicentre NSTE-ACS cohort with long-term follow-up, enabling assessment of both in-hospital and post-discharge HF outcomes. In addition, the use of SHAP-based feature attribution supports transparency and clinical interpretability.

Several limitations should be considered. Heart failure outcomes were clinically defined rather than based on a systematic imaging reference standard. Index-hospitalization HF was adjudicated once per case from medical records, without independent duplicate review or assessment of inter-rater agreement, while post-discharge events were identified using diagnosis codes. Coronary computed tomography angiography scans were acquired for coronary assessment and were not systematically reviewed for HF-specific imaging signs. The VERDICT cohort was a selected trial population with relatively few HF events, including only 22 index-hospitalization events and 39 post-discharge HF hospitalizations, limiting precision, subgroup analyses, and interpretation of diagnostic performance. Only two index-HF events occurred among women, further limiting assessment of performance in women. At the pre-specified threshold, sensitivity and positive predictive value were modest, while the high negative predictive value should be interpreted in light of the low observed HF event rate. NT-proBNP and comprehensive echocardiographic data were not available, and analyses including LVEF were exploratory. More detailed characterization of post-ACS HF risk, including the interplay between HF during the index admission, ventricular dysfunction, and subsequent HF events,^21^ was not possible within the available data and first-event outcome framework.

## Conclusions

We externally validated an interpretable AI algorithm applied to CCTA in patients with NSTE-ACS. Artificial intelligence-derived likelihood stratification identified a large subgroup with low observed HF incidence and a smaller subgroup with increased risk of HF during hospitalization and after discharge. These proof-of-concept findings support further evaluation of CCTA-based AI tools for opportunistic HF assessment in ACS.

## Lead author biography


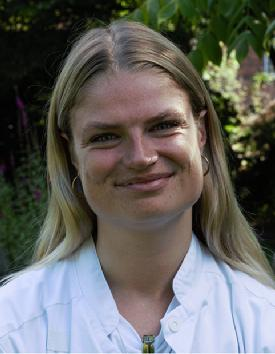
Anne Sophie Overgaard Olesen is a physician and PhD student in cardiology at Copenhagen University Hospital–Bispebjerg and Frederiksberg and the University of Copenhagen, Denmark. She is currently a Visiting Research Fellow at the BHF Cardiovascular Research Centre, University of Glasgow, UK. Her research focuses on improving the diagnosis and treatment of acute and decompensated HF, particularly through clinical trials, diagnostic imaging, and AI-based methods. She also works with meta-analyses and Bayesian approaches to the interpretation of cardiovascular evidence.

## Supplementary Material

ztag133_Supplementary_Data

## Data Availability

The data underlying this article cannot be shared publicly due to the privacy of the individuals who participated in the study. The source code for the AI model is publicly available at https://github.com/orting/breathct. A.S.O.O. had full access to all data in the study and takes responsibility for the integrity of the data and the accuracy of the data analysis.
