# Supplementary material for: Artificial intelligence detection of heart failure on coronary computed tomography angiography: external validation in patients with non-ST-segment elevation acute coronary syndrome
Source: Eur Heart J Digit Health. 2026 Sep 1;7(8):ztag133. doi: 10.1093/ehjdh/ztag133 (PMC13543916; doi:10.1093/ehjdh/ztag133)
Supplement: Supplementary file 1 [file ztag133_supplementary_data.docx]

**Supplementary Tables**

**Supplementary Table 1.** Distribution of heart failure hospitalisations and deaths across AI thresholds

| Threshold | Status | Total | HF during index hospitalisation | HF hospitalisation post-discharge | Total | All-cause mortality during index hospitalisation | All-cause mortality post-discharge | Total |
| --- | --- | --- | --- | --- | --- | --- | --- | --- |
| Rule-in | Positive | 26 | 4 | 1 | 5 | 0 | 6 | 6 |
|  | Negative | 983 | 18 | 38 | 56 | 1 | 99 | 100 |
| Youden | Positive* | 62 | 8 | 3 | 11 | 0 | 15 | 15 |
|  | Negative | 947 | 14 | 36 | 50 | 1 | 90 | 91 |
| Rule-out | Positive | 171 | 12 | 14 | 26 | 0 | 31 | 31 |
|  | Negative† | 838 | 10 | 25 | 35 | 1 | 74 | 75 |

Three diagnostic thresholds were prespecified in the model development cohort to reflect different operating points of the AI algorithm:

- Rule-out threshold: AI score 0.026, with sensitivity ≥90% for CT signs suggestive of heart failure
- Optimal threshold: AI score 0.064, defined using the Youden index
- Rule-in threshold: AI score 0.150, with specificity ≥90%

For the primary analyses, patients were dichotomised using the rule-out threshold to distinguish those with low AI-derived likelihood of heart failure (below rule-out) from those with high likelihood (at or above rule-out threshold).

In supplementary analyses, patients were stratified into three mutually exclusive risk groups using the rule-out and Youden thresholds:

- Low likelihood†: AI score below the rule-out threshold
- High likelihood*: AI score at or above the optimal (Youden) threshold
- Intermediate likelihood: all remaining patients

The rule-in threshold is presented for completeness to illustrate model performance at high specificity but was not used to define likelihood categories in this cohort due to limited event numbers.

**Supplementary Table 2.** Baseline characteristics by heart failure occurrence during the study course

|  | **Missing,**  **n (%)** | **HF during study course**  **(n=61)** | **No HF during study course**  **(N=948)** | ***P* values** |
| --- | --- | --- | --- | --- |
| ***Patient characteristics*** |  |  |  |  |
| Sex male, N (%) | 0 (0) | 45 (74) | 632 (67) | 0.252 |
| Age (years), mean (SD) | 0 (0) | 69.6 (12.0) | 61.3 (11.7) | <0.001 |
| BMI (kg/m2), Median [IQR] | 6 (1) | 27.4 [23.9, 29.4] | 26.3 [24.0, 29.3] | 0.633 |
| History of heart failure, N (%) | 0 (0) | 20 (33) | 75 (8) | <0.001 |
| History of myocardial infarction, N (%) | 0 (0) | 18 (30) | 127 (13) | <0.001 |
| History of diabetes, N (%) | 0 (0) | 13 (21) | 117 (12) | 0.043 |
| History of hypertension, N (%) | 0 (0) | 38 (62) | 449 (47) | 0.024 |
| History of COPD, N (%) | 0 (0) | 13 (21) | 117 (12) | 0.043 |
| History of renal disease, N (%) | 0 (0) | 9 (15) | 51 (5) | 0.008 |
| Current smoker, N (%) | 0 (0) | 17 (28) | 322 (34) | 0.328 |
| ***Admission data*** |  |  |  |  |
| Systolic blood pressure (mmHg), Mean (SD) | 8 (1) | 145 (23) | 141 (22) | 0.197 |
| Heart rate, Median [IQR] | 12 (1) | 78 [68, 90] | 72 [62, 83] | 0.003 |
| Atrial fibrillation at admission, N (%) | 0 (0) | 6 (10) | 27 (3) | 0.012 |
| Increased troponins, N (%)* | 10 (1) | 49 (80) | 732 (77) | 0.231 |
| Creatinine (umol/L), Median [IQR] | 13 (1) | 81 [71, 95] | 75 [66, 87] | 0.005 |
| LVEF at admission (%), Median [IQR] | 141 (14)¶ | 45 [30, 50] | 55 [50, 60] | <0.001 |
| Revascularisation during admission, N (%) | 0 (0) | 35 (57) | 586 (62) | 0.490 |
| Three-vessel or left main coronary artery disease, N (%)‡ | 0 (0) | 21 (34) | 133 (14) | <0.001 |
| GRACE score >140, N (%) | 17 (2) | 45 (74) | 386 (41) | <0.001 |
| Length of index stay, median [IQR]§ | 0 (0) | 2 [1-4] | 2 [1-4] | 0.100 |
| ***Treatment at discharge of admission*** |  |  |  |  |
| Aspirin, N (%) | 4 (0) | 50 (82) | 816 (86) | 0.512 |
| P2Y12 receptor inhibitor, N (%) | 6 (1) | 33 (54) | 675 (71) | 0.006 |
| Oral anticoagulant, N (%) | 5 (1) | 4 (7) | 34 (4) | 0.279 |
| Statin, N (%) | 15 (2) | 53 (87) | 811 (86) | 0.738 |
| Beta-blocker, N (%) | 14 (1) | 50 (82) | 664 (70) | 0.040 |
| ACEi and/or ARB, N (%) | 18 (2) | 36 (59) | 322 (34) | <0.001 |
| Loop-diuretics, N (%) | 15 (2) | 16 (26) | 72 (8) | <0.001 |
| MRA, N (%) | 16 (2) | 8 (13) | 24 (3) | <0.001 |

¶*For LVEF, missing values were similarly distributed between patients with and without heart failure outcome during the study course: 6 of 61 patients (10%) and 135 of 948 patients (14%), respectively.*

*Values are presented as mean (SD), median [IQR], or N (%). Heart failure outcome includes diagnosed heart failure during the index admission or post-discharge heart failure hospitalisation. P values compare patients with and without heart failure outcome during the study course. Treatment variables reflect medication at discharge of the index admission. P2Y12 receptor inhibitor was defined as treatment with clopidogrel, ticagrelor, or prasugrel. ACE inhibitor and/or angiotensin receptor blocker was defined as treatment with an ACE inhibitor, an angiotensin receptor blocker, or both.*

**Supplementary Table 3.** Heart failure-related clinical characteristics across AI HF likelihood groups

|  | **Missing,**  **n (%)** | **Low likelihood (N=838)** | **High likelihood**  **(N=171)** | ***P* values** |
| --- | --- | --- | --- | --- |
| Age (years), mean ± SD | 0 (0) | 61.4 ± 11.8 | 64.1 ± 12.2 | 0.005 |
| LVEF at admission (%), Median [IQR] | 141 (14) | 55 [50, 60] | 55 [45, 60] | <0.001 |
| Increased troponins at admission, N (%)* | 10 (1) | 653 (78) | 128 (75) | 0.382 |
| Three-vessel disease or left main coronary artery disease, N (%)** | 0 (0) | 123 (15) | 31 (18) | 0.253 |
| GRACE score >140, N (%) | 17 (2) | 347 (41) | 84 (49) | 0.050 |
| Heart failure diagnosis day 0-discharge, N (%) | 0 (0) | 10 (1) | 12 (7) | <0.001 |
| Loop diuretic at discharge, N (%) | 15 (2) | 63 (8) | 25 (15) | 0.003 |

**Elevated troponin was defined as > assay-specific upper reference limit, consistent with the VERDICT trial*

***Three-vessel or left main coronary artery disease was assessed by invasive coronary angiography*

**Supplementary Table 4.** Association between three AI-derived likelihood groups and heart failure diagnosis and hospitalisations

| Heart failure diagnosis during index hospitalisation | | | | |
| --- | --- | --- | --- | --- |
| AI HF likelihood | n | HF diagnosis,  n (%) | All-cause mortality  (before HF diagnosis),  n (%) | sHR (95%CI) |
| Low | 838 | 10 (1) | 1 (0) | Ref |
| Intermediate | 109 | 4 (4) | 0 (0) | 3.07  (0.98-9.62) |
| High | 62 | 8 (13) | 0 (0) | 9.48  (3.72-24.20) |
| Heart failure hospitalisation after discharge | | | | |
| AI HF likelihood | n | HF hospitalisation,  n (%) | All-cause mortality  (after discharge, before HF hospitalisation), n (%) | sHR  (95%CI) |
| Low | 838 | 25 (3) | 61 (7) | Ref |
| Intermediate | 109 | 11 (10) | 12 (11) | 3.22  (1.60-6.48) |
| High | 62 | 3 (5) | 14 (23) | 1.45  (0.43-4.93) |

*Low, intermediate, and high AI likelihood were defined using the prespecified rule-out and Youden thresholds. sHRs were estimated using Fine-Gray models with death as a competing event. For post-discharge analyses, patients with heart failure during the index hospitalisation were not at risk for subsequent heart failure hospitalisation but remained under follow-up for mortality.*

**Supplementary Figures**

**Supplementary Figure 1.** Cumulative incidence of all-cause mortality

**Supplementary Figure 2.** Heart failure hospitalisations with death as competing risk

**Supplementary Figure 3.** Cumulative incidence of heart failure hospitalisation according to AI-defined likelihood groups

*Cumulative incidence functions are shown from CCTA to end of follow-up and from hospital discharge to end of follow-up, stratified by low, intermediate, and high AI-defined HF likelihood. Death was treated as a competing event. Numbers below the plots indicate patients at risk.*
